# Covered stent salvage for iatrogenic vertebral artery injury in traumatic cervical spine injury: A case report and literature review

**DOI:** 10.37796/2211-8039.1670

**Published:** 2025-09-01

**Authors:** Tzu-Hao Yen, Wei-Liang Chen, Ying-Lin Tseng, You-Pen Chiu, Hui-Ru Ji, Jeng-Hung Guo, Cheng-Di Chiu

**Affiliations:** a Department of Neurosurgery, China Medical University Hospital, Taichung, Taiwan; b Spine Center, China Medical University Hospital, Taichung, Taiwan; c Stroke Center, China Medical University Hospital, Taichung, Taiwan; d Department of Interventional and Diagnostic Neuroradiology, China Medical University Hospital, Taichung, Taiwan; e School of Medicine, China Medical University, Taichung, Taiwan; f Graduate Institute of Biomedical Science, China Medical University, Taichung, Taiwan

**Keywords:** Cervical spine, Covered stent, Endovascular, Iatrogenic, Vertebral artery

## Abstract

We report the case of a 70-year-old woman who sustained complex traumatic injuries in a motor vehicle accident, including cervical spine fractures and a high suspicion of traumatic vertebral artery injury (VAI). Initial digital subtraction angiography (DSA) revealed no evidence of vertebral artery (VA) involvement. She subsequently underwent anterior cervical discectomy and fusion (ACDF); however, an iatrogenic injury to the right VA occurred intra-operatively, necessitating emergent endovascular stenting for vascular repair. This case underscores the importance of comprehensive preoperative imaging, intraoperative vigilance, and coordinated multidisciplinary management in cervical spine trauma with potential vascular involvement.

## 1. Introduction

Vertebral artery injury (VAI) is a rare but potentially serious complication of cervical spine trauma or surgical procedures [1,2]. Early recognition and appropriate management are crucial to reducing neurological morbidity and preventing ischemic complications. Although uncommon, VAIs are increasingly observed in high-energy cervical spine trauma, particularly in cases involving fractures near the foramen transversarium [3]. We present a case with a high suspicion of traumatic VAI associated with multiple spinal and pelvic fractures; however, an iatrogenic VAI occurred intraoperatively. The patient was successfully treated with a combination of surgical stabilization and endovascular intervention.

## 2. Case report

A 70-year-old woman with a history of hypertension presented to the emergency department following a motorcycle collision with a car. She initially experienced a transient loss of consciousness and was evaluated at a local hospital before being transferred. Upon arrival, she was alert with a Glasgow Coma Scale score of E4V5M6. Physical examination revealed multiple abrasions over the lower extremities, lower abdominal tenderness, and mild bilateral upper limb weakness (muscle strength 4/5) with hyperesthesia. She reported posterior neck pain but no occipital pain.

**Fig. 1:**
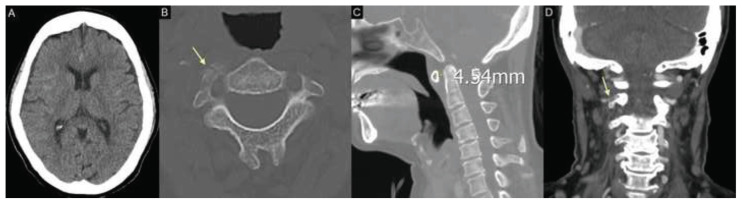
**(A)** Brain computed tomography (CT) demonstrating traumatic subarachnoid hemorrhage. **(B)** Cervical spine CT showing a fracture involving the right C2 pedicle and foraminal wall (yellow arrow). **(C)** Mild widening of the C1–C2 atlantodental interval (ADI: 4.54 mm), suggestive of instability. **(D)** CT angiography demonstrating focal irregular narrowing of the right vertebral artery (VA) at the V3 segment adjacent to the fracture site, raising suspicion of traumatic dissection (yellow arrow).

**Fig. 2:**
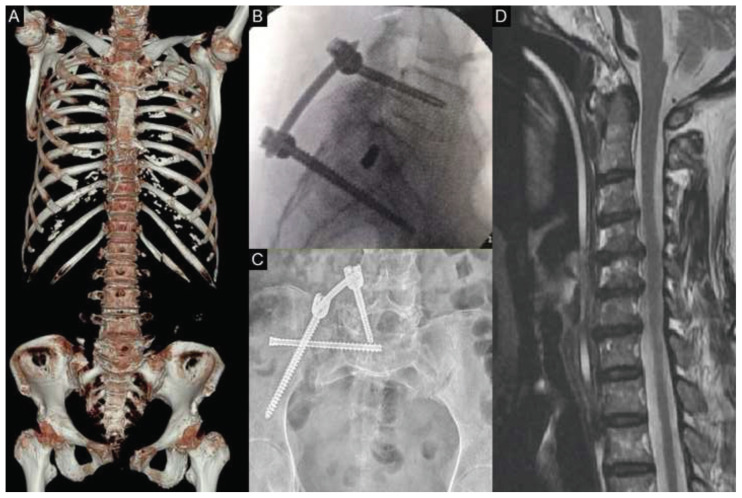
**(A)** Chest–abdomen–pelvis CT showing fractures of the left 4th, 5th, 11th, and 12th ribs, and complex pelvic fractures involving the right sacrum, right iliac wing, and bilateral pubic rami. **(B)** Intraoperative sagittal fluoroscopic view demonstrating placement of right L5-S2-alar-iliac screw fixation (6.5 × 50 mm and 7.5 × 90 mm). **(C)** Postoperative pelvic radiograph showing stabilization of the sacral fracture with a 7.0 × 90 mm Aspire headless screw. **(D)** Sagittal magnetic resonance imaging (MRI) of the cervical spine demonstrating pronounced multilevel bilateral foraminal stenosis from C4 to C7.

A brain computed tomography (CT) scan showed a mild traumatic subarachnoid hemorrhage (Fig. 1A). Cervical spine CT revealed a fracture involving the right C2 pedicle and foraminal wall (Fig. 1B), along with mild widening of the C1–C2 atlantodental interval (ADI: 4.54 mm), suggestive of segmental instability (Fig. 1C). Computed tomography angiography (CTA) of the cervical spine demonstrated focal irregular narrowing of the right VA at the V3 segment adjacent to the fracture site, raising suspicion for traumatic dissection (Fig. 1D). Additional chest-abdomenpelvis CT imaging revealed fractures of the left 4th, 5th, 11th, and 12th ribs, along with complex pelvic injuries involving the right sacrum, right iliac wing, and bilateral pubic rami—consistent with an AO/OTA type II acetabular fracture (Fig. 2A). Magnetic resonance imaging (MRI) of the cervical spine demonstrated multilevel spinal stenosis from C4 to C7 with significant bilateral foraminal narrowing (Fig. 2D). The patient was admitted to the neurosurgical intensive care unit for close monitoring and further management.

Initial treatment included thoracolumbosacral orthosis bracing and right L5-S2-alar-iliac fixation using 6.5 × 50 mm and 7.5 × 90 mm screws (Fig. 2B). To stabilize the sacral fracture, a closed reduction and internal fixation was performed using a 7.0 × 90 mm Aspire headless screw (Fig. 2C). Intraoperative fluoroscopy confirmed satisfactory alignment and stability of the fixation.

Given the suspected right VAI identified on initial CTA, preoperative digital subtraction angiography (DSA) was performed, which revealed no significant stenosis or dissection. Due to the underlying cervical spine pathology and persistent symptoms, anterior cervical discectomy and fusion (ACDF) from C4 to C7 was performed. Intraoperatively, a large central ruptured disc and osteophytic spurs were observed at the C4/5 level, along with severe foraminal stenosis. Additional disc herniation and prominent osteophytes were identified at the C5/6 and C6/7 levels. During decompression at C6/7, profuse and brisk bleeding from the right uncinate process was encountered, raising concern for a possible iatrogenic injury to the right VA. Immediate tamponade was applied using Gelfoam®, shaped into small peanut-sized pieces approximately 0.5 cm in diameter. A total of 3–4 pieces were applied to control bleeding while maintaining partial arterial flow. The estimated intraoperative blood loss was approximately 2900 ml.

**Fig. 3:**
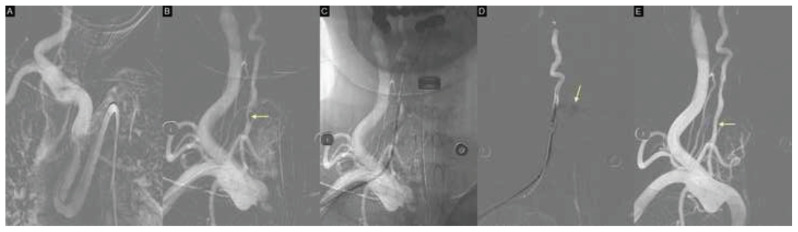
**(A)** Digital subtraction angiography (DSA) with roadmap imaging demonstrating a type III aortic arch. **(B)** Severe luminal narrowing of the right VA at the C6/7 level (yellow arrow). **(C)** A 200-cm Boston V-18™ ControlWire (0.018-inch) microwire advanced across the stenotic segment, with a 5-Fr, 125-cm JB2 catheter navigated into the right vertebral artery up to the C3/4 level. **(D)** Pre-deployment angiography showing contrast extravasation at the injury site (yellow arrow). **(E)** Post-deployment angiography after placement of a 5 mm × 5 cm VIABAHN® covered stent graft, showing resolution of contrast leakage (yellow arrow), immediate hemostasis, and restoration of vessel patency.

Because of the presence of a type III aortic arch (Fig. 3A), catheter navigation to the right subclavian artery via the right femoral approach was challenging. Emergency endovascular repair was subsequently performed through a 7-Fr right brachial artery sheath. Roadmap imaging of the right subclavian artery using a 5-Fr, 125-cm JB2 catheter confirmed a right VA injury, characterized by focal severe stenosis, luminal irregularity, and minimal contrast extravasation at the C6/7 level (Fig. 3B). A 200-cm Boston V-18™ ControlWire (0.018-inch) microwire was positioned across the stenotic segment, after which the 5-Fr, 125-cm JB2 catheter was navigated into the right VA up to the C3/4 level (Fig. 3C). After confirming the proximal and distal vessel diameters and lesion length, a 5 mm × 5 cm VIABAHN® covered stent was successfully deployed. Pre-deployment angiography showed contrast leakage (Fig. 3D), which resolved following stent placement. Post-stenting roadmap imaging confirmed complete sealing of the injury and restoration of antegrade flow (Fig. 3E). The brachial access site was closed with manual compression.

Antiplatelet therapy was initiated with a loading dose of clopidogrel 150 mg every 12 h for three doses, followed by clopidogrel 75 mg daily for six months. This was later transitioned to aspirin 100 mg daily for an additional six months.

**Fig. 4:**
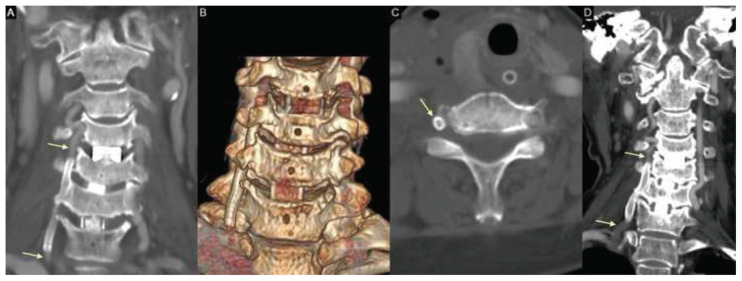
**(A)** Postoperative contrast-enhanced cervical spine CT on postoperative day 3 confirming stent patency with preserved flow through the right VA (yellow arrow). **(B)** 3D reconstruction of the cervical spine illustrating the anatomical relationship between the covered stent and the cervical foramina. **(C)** Axial cervical spine CT showing the position of the covered stent adjacent to the uncinate process (yellow arrow). **(D)** Contrast-enhanced cervical spine CT obtained one year later demonstrating persistent patency of the right VA across the stented segment (yellow arrow).

A contrast-enhanced cervical spine CT performed three days postoperatively confirmed stent patency and preserved flow in the right VA (Fig. 4A–C). Postoperative brain MRI revealed no evidence of acute infarction in the posterior circulation. The patient remained neurologically intact and was discharged on postoperative day 7. During outpatient follow-up, she remained stable. One year later, repeat contrast-enhanced cervical spine CT demonstrated persistent patency of the right VA without evidence of in-stent restenosis (Fig. 4D). Antiplatelet therapy was subsequently discontinued.

## 3. Discussion

Vertebral artery injury (VAI), particularly when associated with cervical spine trauma or surgery, presents a critical and complex clinical challenge. Although preoperative imaging—such as computed tomography angiography (CTA), magnetic resonance angiography (MRA), or digital subtraction angiography (DSA)—can assist in identifying stenosis, dissection, or anatomical variations, iatrogenic VAI may still occur intraoperatively despite thorough evaluation [1,4,5]. This is largely due to the close anatomical relationship between the uncinate process and the foramen transversarium, placing the VA at particular risk during anterior cervical procedures— especially in multilevel discectomies or in patients with atypical anatomy [3,6].

VAI resulting from blunt cervical trauma also poses significant diagnostic and management challenges [5]. In the present case, traumatic displacement near the C2 pedicle could have led to vascular compromise, although no radiographic abnormality was initially detected on angiographic studies. Notably, even though preoperative DSA did not reveal significant stenosis or dissection, the intraoperative iatrogenic injury highlights a key consideration: negative preoperative imaging does not eliminate the risk of VA injury, particularly in patients with unstable fractures, fracture fragments adjacent to the transverse foramen, or aberrant vascular courses [2,7,8].

Intraoperative VAI is rare, with an estimated incidence of 0.2 %–0.5 % in anterior cervical procedures [8–11]. Lee et al. analyzed data from 21 multicenter studies and reported an overall VAI incidence of 0.08 %, with the highest risk associated with C1–C2 posterior fixation (1.35 %), followed by C3–C6 levels (0.2 %) [12]. Similarly, Akinduro et al. conducted a systematic review and reported a VAI incidence of 2.9 % in posterior C1–C2 fixation [13]. Common causes include screw misplacement, high-speed drilling, and soft tissue retraction, as summarized in Table 1 [5, 915]. The V2 segment of the VA—running through the transverse foramina from C6 to C2—is most frequently involved due to its anatomical fixation and proximity to the surgical field. Risk factors include fracture extension into the foramen transversarium, congenital anatomy anomalies, and distorted landmarks from trauma. When VAI occurs, it typically presents as sudden, forceful arterial bleeding requiring immediate tamponade. It carries significant risks, including pseudoaneurysm formation, ischemic stroke, and potentially death. Definitive treatment depends on the patient’s hemodynamic stability and institutional resources, with common options including endovascular coil embolization, stenting, and, less frequently, surgical repair or ligation [5,9–17]. Early recognition and prompt interdisciplinary intervention are critical to minimizing morbidity.

Multiple studies have emphasized the importance of early vascular evaluation in high-risk cervical injuries, recommending prompt intervention when vascular compromise is suspected [2,5,16]. In select cases, some authors have advocated for preoperative endovascular stenting in patients with suspected VA involvement to minimize the risk of intraoperative hemorrhage [10,16,17]. However, the decision to prioritize spinal decompression should be driven by clinical urgency, including progressive neurological symptoms, spinal instability, and overall risks associated with surgical intervention. Additionally, intraoperative preparedness for hemostatic control and close collaboration with interventional specialists remain critical [10,17].

The decision to sacrifice a VA should be made with great caution. The success of unilateral. VA occlusion depends on sufficient collateral circulation from the contralateral VA to maintain the perfusion of the basilar artery. Inadequate compensation can lead to serious neurological complications—such as brainstem or cerebellar infarction, cranial nerve palsies, or hemiplegia, with reported mortality rates up to 12 % [18]. Therefore, preserving VA flow is important, even in hypoplastic arteries, as the long-term consequences of occlusion remain incompletely understood.

Endovascular intervention has become the cornerstone of VAI management, providing both high-resolution diagnostic capability and therapeutic efficacy. DSA offers superior spatial and temporal resolution, allowing detailed visualization of vessel wall integrity, subtle dissection, and extravasation—findings that are often missed on CTA or MRA [2,7]. Importantly, endovascular procedures allow immediate treatment within the same session, including coil embolization or covered stent deployment, thereby minimizing delays and reducing risk. In cases where vascular injury is suspected either preoperatively or intraoperatively, the timely application of endovascular techniques enables rapid, life-saving hemorrhage control [16,17].

In our institution, two types of covered stents were available: VIABAHN® and Biotronik®. After measuring the injured VA diameter, we selected the VIABAHN® covered stent for its optimal fit and nearly complete mesh coverage, minimizing blood leakage. The stent was successfully deployed intraoperatively, effectively controlling hemorrhage and restoring vessel patency, which allowed completion of the anterior decompression and fusion. Antiplatelet therapy was initiated with clopidogrel 150 mg every 12 h for three doses, followed by 75 mg daily for six months, and then transitioned to aspirin 100 mg daily for another six months to prevent in-stent thrombosis and maintain patency [17,19,20].

This case underscores the importance of thorough preoperative vascular assessment in patients with upper cervical fractures [7]. The decision to proceed with anterior decompression was driven by progressive neurological deficits and structural instability. Intraoperative suspicion of VAI led to immediate endovascular repair with a covered stent, enabling the safe continuation of the surgical procedure [19]. Postoperative management included tailored antiplatelet therapy to ensure long-term graft patency and prevent thrombotic complications. The patient’s favorable recovery highlights the effectiveness of a multidisciplinary approach involving spine surgeons, interventional radiologists, and critical care teams.

This case underscores the critical need for heightened vigilance regarding vertebral artery injury in cervical spine trauma. Timely diagnosis and coordinated interventions, such as endovascular stenting, are essential to minimizing the risk of severe vascular complications and optimizing surgical outcomes. In patients with high-risk cervical injuries, surgeons should maintain a high index of suspicion and incorporate vascular imaging as a routine component of preoperative planning.

## Figures and Tables

**Table 1:** Summary of iatrogenic vertebral artery injury.

Author	Procedure	Inclusion	Incidence	Mechanism	Management	Outcome
Burke et al., 2005 [10]	Anterior	A large center, 1994–2001, retrospective	0.3 % (6/1976)	Not specified in detail	Tamponade (3 cases), direct repair/ligation (3 cases)	Two complications in tamponade group; none in repair group
Neo et al., 2008 [9]	Anterior & Posterior	5641 cases, retrospective	Overall, 0.14 % (8/5641) (anterior: 0.18 %; posterior C1–C2: 1.3 %)	Screw misplacement; Drilling	Tamponade, embolization	No deaths; inexperienced surgeons had higher risk
Molinari et al., 2014 [11]	Anterior & Posterior	72 citations up to 2013/4, systematic review	0.20 ~ 1.96 %	Not specified in detail	Tamponade, direct repair/ligation, or embolization	Primary repair/ligation success 100 %; tamponade less reliable
Akinduro et al., 2016 [13]	Posterior C1–C2	27 publications, systematic review	2.9 % (60/2078)	Screw misplacement (21.7 %); Drilling (15.0 %); Exposure (8.3 %); Tapping (8.3 %); Unknown (46.7 %)	Tamponade, repair, or embolization	Ipsilateral stroke (10 %); permanent deficits (1.7 %); arteriovenous fistula (8.3 %); pseudoaneurysm (3.3 %); mortality (6.7 %)
Guan et al., 2017 [5]	Anterior	25 studies, 1980–2017, systematic review	54 cases (10 patients had VA anomalies)	Drilling (61 %); Instrumentation (16 %); Retraction (8 %)	Tamponade, repair, or embolization	Pseudoaneurysm in 48 % after tamponade; neurologic sequelae in 41 % if collateral status unknown; repair/stent had excellent prognosis
Lee et al., 2019 [12]	Posterior C1–C6	14,722 cases in 21 centers, 2012–2016, retrospective	Overall, 0.08 % (C1–C2: 1.35 %; C3–C6: 0.20 %)	Screw misplacement (31 %); High-speed drilling (23 %)	Tamponade, repair	23 % had cerebellar or brainstem infarction
Turgut et al., 2022 [14]	Anterior & Posterior	72 articles, 1962–2021, systematic review	194 cases (16 patients had VA anomalies)	Screw misplacement (31.4 %); Drilling (20.6 %)	Tamponade, direct repair/ligation, or embolization	Laceration (41.2 %); pseudoaneurysm (16.5 %); dissection (5.7 %); Mortality (7.2 %)
Barrie et al., 2023 [15]	Anterior	43 articles, systematic review	75 cases	Drilling (40 %); Instrumentation (13 %)	Tamponade, direct repair/ligation, or embolization	Majority recovered without major deficits
